# Expression and Characterization of an Efficient Alginate Lyase from *Psychromonas* sp. SP041 through Metagenomics Analysis of Rotten Kelp

**DOI:** 10.3390/genes15050598

**Published:** 2024-05-08

**Authors:** Ping Wang, Yi Cai, Hua Zhong, Ruiting Chen, Yuetao Yi, Yanrui Ye, Lili Li

**Affiliations:** 1 Qingdao Academy of Chinese Medical Sciences, Shandong University of Traditional Chinese Medicine, Qingdao 266112, China; wangpingdoct@163.com; 2 Yantai Institute of Coastal Zone Research, Chinese Academy of Sciences, Yantai 264003, China; ytyi@yic.ac.cn; 3 School of Biology and Biological Engineering, South China University of Technology, Guangzhou 510006, China; 202110189261@mail.scut.edu.cn (Y.C.); 202221049742@mail.scut.edu.cn (R.C.); 4 School of Pharmacy, Binzhou Medical University, Yantai 264003, China; 18279017719@163.com

**Keywords:** alginate lyase, alginate oligosaccharide, metagenomic analysis, brown seaweed

## Abstract

Alginate is derived from brown algae, which can be cultivated in large quantities. It can be broken down by alginate lyase into alginate oligosaccharides (AOSs), which exhibit a higher added value and better bioactivity than alginate. In this study, metagenomic technology was used to screen for genes that code for high-efficiency alginate lyases. The candidate alginate lyase gene *alg169* was detected from *Psychromonas* sp. SP041, the most abundant species among alginate lyase bacteria on selected rotten kelps. The alginate lyase Alg169 was heterologously expressed in *Escherichia coli* BL21 (DE3), Ni-IDA-purified, and characterized. The optimum temperature and pH of Alg169 were 25 °C and 7.0, respectively. Metal ions including Mn^2+^, Co^2+^, Ca^2+^, Mg^2+^, Ni^2+^, and Ba^2+^ led to significantly increased enzyme activity. Alg169 exhibited a pronounced dependence on Na^+^, and upon treatment with Mn^2+^, its activity surged by 687.57%, resulting in the highest observed enzyme activity of 117,081 U/mg. Bioinformatic analysis predicted that Alg169 would be a double-domain lyase with a molecular weight of 65.58 kDa. It is a bifunctional enzyme with substrate specificity to polyguluronic acid (polyG) and polymannuronic acid (polyM). These results suggest that Alg169 is a promising candidate for the efficient manufacturing of AOSs from brown seaweed.

## 1. Introduction

Brown algae are sustainable and renewable resources that provide rich bioactive carbohydrate compounds, including polysaccharides and oligosaccharides [1]. Alginate is a linear polysaccharide found in the cell walls of brown algae, such as kelp and sargassum, formed by the connection of *β*-D-mannuronic acid (M) and *α*-L-guluronic acid (G) through 1,4-glycosidic bonds [2]. These basic units are arranged in different forms to compose three types of blocks, including poly *α*-L-guluronic acid (polyG), poly *β*-D-mannuronic acid (polyM), and their hybrids (polyGM), in which the monomers are connected by *α*-(1,4) glycosidic bonds [3]. Alginate oligosaccharides (AOSs) are molecules that result from the breakdown of alginate. AOSs have higher bioavailability, higher added value, and better biological activity than alginate. They exhibit various effects such as anti-tumor [4], anti-diabetes [5], anti-inflammatory [6], neuroprotection [7], immune regulation [8], and antioxidation [9] effects. For example, we previously found that AOSs regulate antibiotic-induced microbial disorders and improve obesity-related outcomes [10,11].

Various methods, including physical, chemical, and biological approaches, are commonly employed in the preparation of alginate oligosaccharides [12]. Physical methods typically encompass radiation [13], thermal degradation [14], and ultrasound [15]. These methods exhibit high energy consumption and can easily result in the inhomogeneity and incomplete degradation of pyrolysis products. Chemical methods include hydrochloric acid [16] and oxidative digestion [17]. They are often associated with serious pollution. Biological methods are renowned for their lower energy consumption, environmental friendliness, lower generation of byproducts, and mild reaction conditions [18,19]. There are two biological methods for the preparation of AOSs from alginate. One method utilizes alginate-lysing microorganisms, particularly bacteria, while the other employs alginate lyase. The enzymatic approach exhibits several advantages over the microorganism approach. For example, it enables the precise targeting and elimination of the 1–4 glycosidic bond within alginate molecules, resulting in clear product structures with exceptional purity that facilitate subsequent separation and purification procedures. In contrast, the microorganism approach involves intricate biological pathways that can introduce variability, potentially compromising product quality and purity [20].

Cloning alginate lyase from alginate lyase-containing bacterium is the traditional screen method. It needs to filter bacteria with secretory-alginate lyase first, then identify whether it is a novel alginate-degrading bacteria through 16S sequencing, so it always discovers novel alginate-degrading bacteria but easily misses those strains with the inclusion-alginate lyase. Some bacterium can only be cultured at special temperatures, and with carbon and nitrogen sources, which also limits the result of screening alginate lyase [21]. Direct cloning and expression of alginate lyase in existing gene libraries is a convenient method, but new computational methods still need to be developed to improve the efficiency of screening specific enzymes [22]. Though using metagenomic methods to sequence environmental microorganisms and formulating a screening criterion, with the addition of bioinformatics analyses, screening for alginate lyase genes can avoid this defect.

In the development of screening criteria, lyases with low sequence similarity can be highly meaningful enzymes. However, their activity can vary significantly, leading to challenges in consistently identifying candidates with high enzymatic efficiency. In our study, we considered the high abundance of bacteria as an indicator of their potential for robust growth and efficient alginate degradation. This approach allows us to prioritize enzymes that are not only prevalent within their respective microbial communities, but which are also likely to exhibit efficient substrate degradation capabilities.

Alginate lyases (EC4.2.2.-) can specifically degrade alginate into active AOSs. They mainly eliminate the 1–4 glycosidic bond of alginate by *β*-elimination reaction and generate unsaturated uronic acid with C4–5 double bond at the non-reducing end [23]. Alginate lyases exhibit a wide range of sources, including bacteria, fungi, viruses, and mollusks [24]. According to the carbohydrate activity (CZAy) database http://www.cazy.org/ (accessed on 9 December 2023), alginate lyases are included in 14 polysaccharide lyase families (PL5, 6, 7, 8, 14, 15, 17, 18, 31, 32, 34, 36, 39, and 41) [25].

Until now, a variety of alginate lyases from marine microorganisms have been reported [26,27,28]. In this study, a novel alginate lyase, Alg169, was identified through a metagenomics approach from the bacterium *Psychromonas* sp. SP041, which was found on the surface of refrigerated rotten kelps. Alg169 was subsequently overexpressed and characterized. The findings of this study highlight the successful acquisition of an effective alginate lyase for producing AOSs.

## 2. Materials and Methods

### 2.1. Materials

Sodium alginate was obtained from Sinopharm Chemical Reagent Co., Ltd. (Shanghai, China). PolyG and polyM were purchased from Qingdao Hehai Biotechnology Co., Ltd. (Qingdao, China). *E. coli* BL21 (DE3)-competent cells and the Bradford protein quantification assay kit were bought from Beijing Solarbio Technology Co., Ltd. (Beijing, China). BeaverBeads^®^ His-tag Protein Purification IDA-Nickel magnetic beads were purchased from Suzhou Beaver Biomedical Engineering Co., Ltd. (Suzhou, China). SurePAGE™ protein precast gels were obtained from Nanjing GenScript Biotechnology Co., Ltd. (Nanjing, China). Silica gel 60 F254 thin-layer chromatography (TLC) plates and Amicon^®^ Ultra-15 ultrafiltration centrifuge tubes were obtained from Merck KgaA (Darmstadt, Germany).

### 2.2. Metagenomic Screening and Cloning of Alginate Lyase Gene

Kelp samples were collected from Changdao Island (China) and sargassum samples were collected from Yangma Island (China); both were stored at 4 °C for 15 days. The bacterium samples were collected from the surface of placed rotted kelp, from the liquid cultured rotted kelp, and from the liquid cultured rotted sargassum; these were named HDM01, HDJ02, and SWZ03, respectively. Then, metagenomic sequencing of the kelp samples was performed by Novogene Technologies Co., Ltd. (Tianjin, China). Three strains containing the alginate lyase gene with the highest absolute abundance were screened from the metagenomic data, and four genes containing the alginate lyase conserved domain sequence in them detected by SMART https://smart.embl.de/ (accessed on 20 September 2023), were selected for further study. Then, we used blastX of NCBI https://www.ncbi.nlm.nih.gov/ (accessed on 20 September 2023), to compare the similarity of the four candidate amino acid sequences with the amino acid sequence of the known reference enzymes. They have high similarity to the amino acid sequence of the known reference enzymes, but none of them have been studied for the activity of alginate degradation. After removing the signal peptide http://www.cbs.dtu.dk/services/SignalP/ (accessed on 22 September 2023), and performing codon optimization https://www.genscript.com.cn/ (accessed on 24 September 2023), the genes were sent to Qingke Biotechnology Co., Ltd. (Tianjin, China) for synthesis.

### 2.3. Structure Prediction of Alg169

The sequence of Alg169 is shown in the Supplementary Materials as Table S1. Alg169 was aligned with the NR (non-redundant) protein database using NCBI BLAST https://blast.ncbi.nlm.nih.gov/Blast.cgi (accessed on 8 December 2023). Its theoretical isoelectric point (pI) and molecular weight (MW) were determined via the ProtParam tool of Expasy https://web.expasy.org/protparam (accessed on 8 December 2023) [29]. Then, we made the signal peptide predictions using SignalP 5.0 https://services.healthtech.dtu.dk/services/SignalP-5.0 (accessed on 8 December 2023) [30]. The search for conserved domains within Alg169 was conducted through a Conserved Domain search of NCBI https://www.ncbi.nlm.nih.gov/Structure/cdd/wrpsb.cgi (accessed on 8 December 2023) [31]. The phylogenetic lineage of Alg169 was delineated using MEGA X v10.0.5. Furthermore, the 3D conformation of Alg169 was predicted utilizing AlphaFold2 [32] with default settings, and its structural quality was evaluated using the SAVES v6.0 https://saves.mbi.ucla.edu (accessed on 9 December 2023). The binding affinities between Alg169 and its specific substrates were predicted through molecular docking executed by Autodock Vina 1.2.5, with the docking results visualized using PyMOL 2.5.

### 2.4. Expression and Purification of Alg169

The synthesized genes (*alg169*, *alg179*, *alg189*, and *alg136*) were cloned into pET21a transformed into pET21a (+), and expressed by *E. coli* BL21 (DE3); the gene sequences are provided in Supplementary Materials Table S1. Transformants were verified by polymerase chain reaction (PCR) using primers pET21a-F and Alg169-R (Alg169 amplification and identification primers are listed in Supplementary Materials Table S2). Positive transformants were cultured in LB liquid medium with 100 μg/mL ampicillin until the OD_600_ was about 0.6–0.8. To induce the expression of Alg169, isopropyl-*β*-D-thiogalactopyranoside (IPTG) was added at a final concentration of 0.5 mmol/L and the recombinant *E. coli* cells were further cultured at 16 °C for 16 h. *E. coli* cells were collected by centrifugation, washed twice with 10 mmol/L PBS (pH 7.4), suspended in lysis buffer (20 mmol/L PBS, 500 mmol/L NaCl, 20 mmol/L imidazole, pH 7.4), and then lysed by sonication. The supernatant of cell lysis was purified and concentrated by His-tag Protein Purification IDA-Nickel magnetic beads. The beads were initially equilibrated with binding buffer (20 mmol/L PBS, 500 mmol/L NaCl, 20 mmol/L imidazole, pH 7.4) before removing impurity proteins with wash buffer (20 mmol/L PBS, 500 mmol/L NaCl, 50 mmol/L imidazole, pH 7.4). Alg169 was further washed with elution buffer (20 mmol/L PBS, 500 mmol/L NaCl, 500 mmol/L imidazole, pH 7.4), concentrated using Amicon^®^ Ultra-15 ultrafiltration centrifuge tubes, and analyzed by Sodium dodecyl sulfate-polyacrylamide gel electrophoresis (SDS-PAGE) and liquid chromatography–tandem mass spectrometry (LC-MS/MS). Protein concentration was determined using a Bradford protein quantification assay kit.

### 2.5. Enzyme Activity Assay

To measure enzyme activity, 0.1 mL of the diluted enzyme solution was mixed with 0.9 mL of 50 mmol/L Tris-HCl buffer (pH 7.4) containing 10 g/L sodium alginate. The mixture was incubated at 25 °C for 20 min. Then, 1.5 mL of DNS reagent was added to the mixture and incubated in boiling water for 3 min to stop the enzymatic hydrolysis reaction. Enzyme activity was determined by measuring the concentration of reducing sugar through enzyme calibration (Flash Biotechnology Co., Ltd., Shanghai, China) at the absorbance of 540 nm (A_540_). One unit (U) is defined as the amount of enzyme that degrades sodium alginate to produce 1 μg of reducing sugar per minute (25 °C, 50 mmol/L Tris-HCl buffer containing 1% sodium alginate, pH 7.0) [33].

### 2.6. Characterization of Recombinant Alg169

The effect of temperature on Alg169 activity was determined at 20 °C, 25 °C, 30 °C, 35 °C, 40 °C, and 45 °C. Alg169 was incubated for two hours; then, the remaining enzyme activity was detected to determine the temperature stability of Alg169. The effect of pH on Alg169 activity was calculated at pH values of 4.0, 5.0, 6.0, 7.0, 8.0, 9.0, 10.0, and 11.0. The pH value of the reaction system was adjusted by a buffer containing 50 mmol/L Na_2_HPO_4_-citric acid (pH 4.0–6.0), 50 mmol/L Tris-HCl (pH 7.0–9.0), and 50 mmol/L glycine-NaOH (pH 10.0–11.0). The pH stability of Alg169 was investigated at these pHs with 5 mmol/L Mn^2+^ supplemented at 4 °C. Residual enzyme activity was measured after 24 h. The best enzyme activity without place treatment was used as a control. To explore the effect of metal ions, enzyme activity was assayed by incubating K^+^, Na^+^, Mg^2+^, Ca^2+^, Fe^3+^, Cu^2+^, Zn^2+^, Ni^2+^, Mn^2+^, Ba^2+^, Co^2+^, and EDTA with Alg169 at a final concentration of 5 mmol/L for 18 h. Moreover, the activity of Alg169 was estimated at 0, 50, 100, 150, 200, and 250 mmol/L NaCl concentrations to inquire about the effect of NaCl concentration on Alg169. The kinetic parameters were measured in Tris-HCl buffer (50 mmol/L, pH 7.0) with 5 mmol/L Mn^2+^ and 150 mmol/L NaCl at 25 °C; the concentration of sodium alginate was set to 1, 2, 4, 6, 8, and 10 mg/mL. PolyG and polyM were also determined using the same system [26]. The enzymatic kinetic parameters K_m_ and k_cat_ were calculated by the Michaelis–Menten equation using GraphPad Prism 9.5.0 software.

### 2.7. Analysis of the Enzymatic Hydrolysis Products of Alginate

Sodium alginate at a concentration of 10 g/L was incubated with 0.465 mg of purified recombinant Alg169. The reaction was carried out at 25 °C for 1 min, 5 min, 10 min, 15 min, 20 min, 1 h, 6 h, 12 h, 24 h, 48 h, and 72 h. Subsequently, aliquots from each time point were spotted on the TLC plates and developed using a mixture of n-butanol/formic acid (98%)/water (4:5:1 *v*:*v*:*v*). The plates were then placed vertically in a sealed chamber, sprayed with a solution containing 10% (*v*/*v*) sulfuric acid in ethanol, and heated at 95 °C for 30 min to visualize the enzymatic degradation products.

## 3. Results

### 3.1. Metagenomic Data Analysis

**Figure 1:**
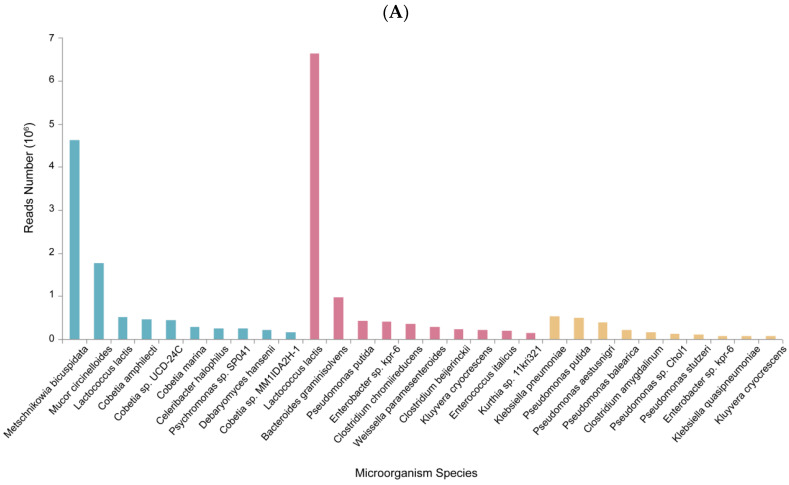

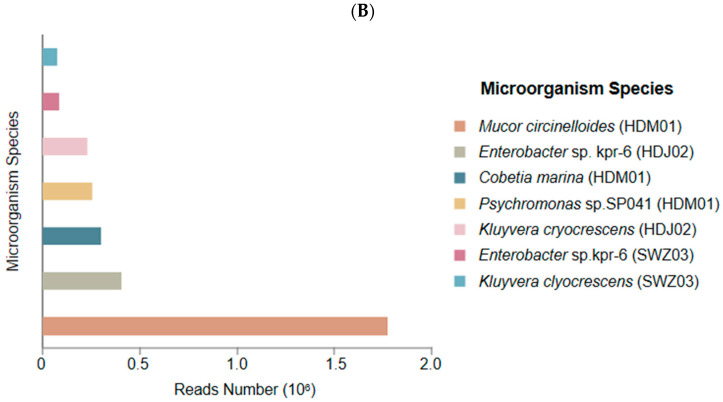
Metagenomic data analysis and ranking of alginate lyase genes abundance. (**A**) The top-ten species in the absolute abundance of the three samples were HDM01 (in green), HDJ02 (in red), and SWZ03 (in yellow). (**B**) Comparison of the absolute abundance of the top-ten alginate lyase-containing species.

The top-ten most abundant species in each sample were identified through metagenomic sequencing (Figure 1A) and then analyzed by aligning the sequences on the CAZy database. Only five of the top-ten strains in the absolute abundance of each sample contained the genes with alginate lyase screened from the CAZy database, which were *Mucor circinelloides*, *Cobetia marina*, *Psychromonas* sp. SP041 in HDM01, *Enterobacter* sp. kpr-6, and *Kluyvera cryocrescens* in HDJ02 and SWZ03, respectively (Figure 1B). The alginate lyase genes in each strain were selected from the Unigene database and sorted according to the absolute abundance of the strains from high to low. Following the SMART identification approach, four alginate lyase sequences were identified with a conserved alginate lyase domain. These sequences ranked among the top three in terms of absolute abundance within the following strains: HDJ02-189966 from *Enterobacter* sp. kpr-6, HDM01-179661 from *Cobetia marina*, and HDM01-16965 as well as HDM01-13691 from *Psychromonas* sp. SP041. The simplified names are *alg189*, *alg179*, *alg169*, and *alg136*. These four genes were synthesized and expressed.

### 3.2. Sequence Analysis and Molecular Docking of Alg169

**Figure 2:**
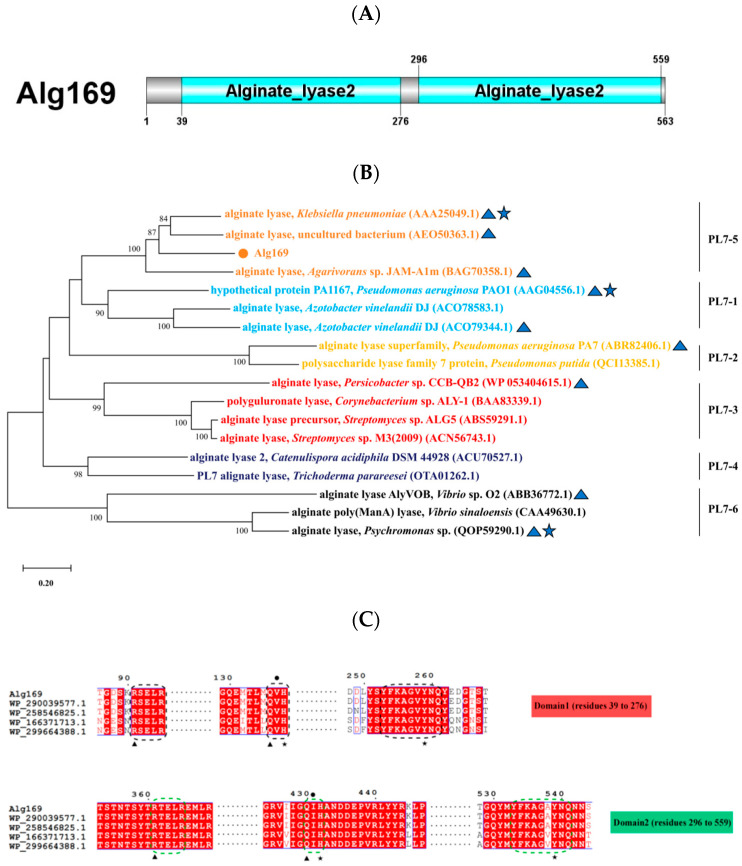
(**A**) Schematic representation of Alg169 domain configuration. (**B**) The phylogenetic tree of Alg169 and other alginate lyases within the PL7 family was constructed using MEGA X. Bootstrap values for each branch were obtained through 1000 repetitions. Alg169 is indicated by a spot, the characterized alginate lyases are shown by triangles, and known structure alginate lyases are marked with pentagrams. (**C**) Result of alignment analysis of Alg169 and related alginate lyases (WP_290039577.1, alginate lyase of *Psychromonas* sp. 14N.309.X.WAT.B.A12; WP_258546825.1, alginate lyase of *Psychromonas* sp. B3M02; WP_166371713.1, alginate lyase of *Psychromonas* sp. SA13A; and WP_299664388.1, alginate lyase of uncultured *Psychromonas* sp.). The black and green dashed boxes indicate the conserved motifs for the catalytic activity of the PL7 family in Domain 1 and Domain 2, respectively. Neutralizing residues and catalytic acids/bases are marked with black triangles and stars. Black circles pinpoint amino acids related to catalytic activity. Amino acids that are conserved across the sequences are highlighted with a red background and white character.

**Figure 3:**
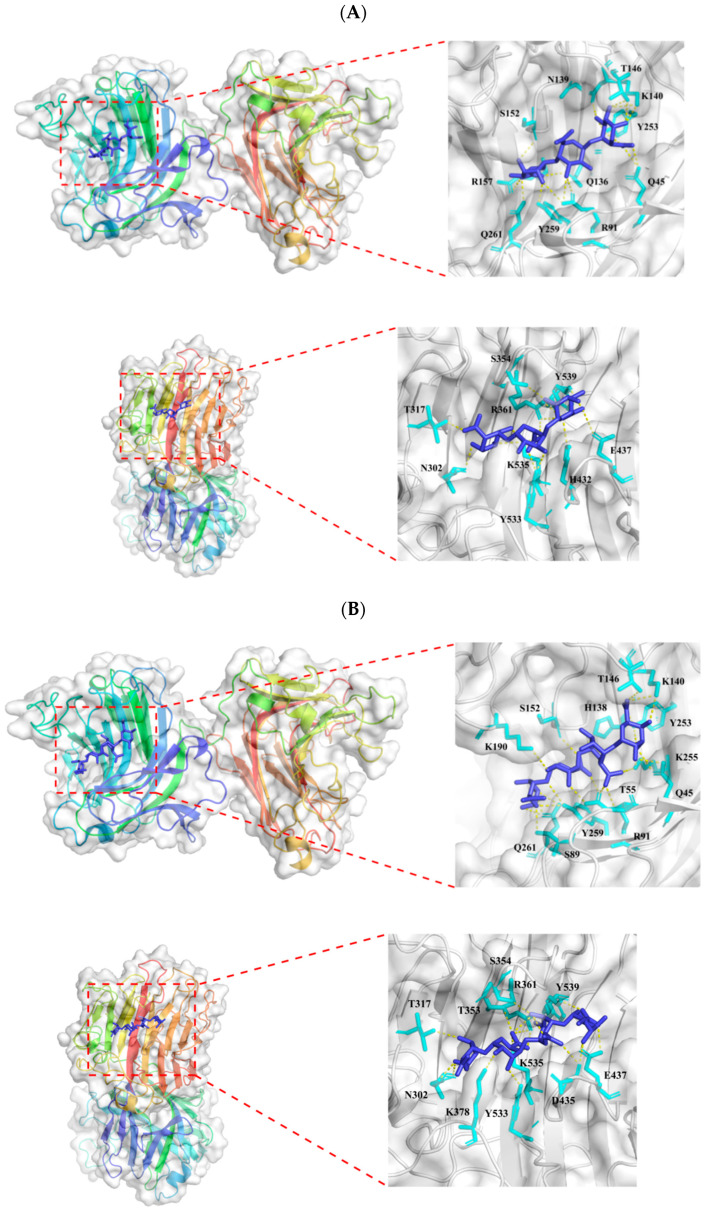

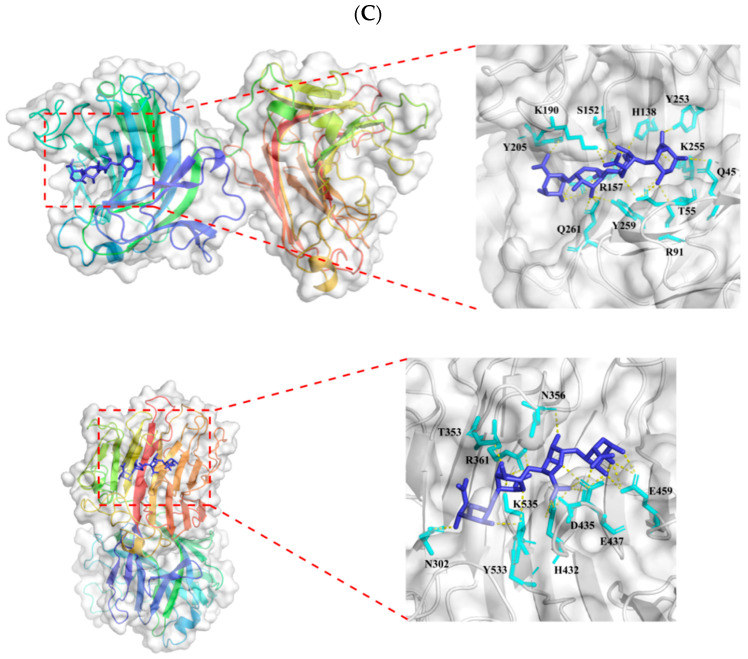
Molecular docking analysis of Alg169. Binding sites and docked poses of (**A**) alginate, (**B**) L-tetraguluronic acid, and (**C**) D-tetramannuronic acid are shown in the three panels, respectively. For each panel, the upper section shows the docking result for the ligand with Domain 1 of Alg169, and the lower section shows the docking result for the ligand with Domain 2 of Alg169. A detailed view highlighting the residues forming polar contacts with the ligand is shown on the right for each section. The protein structure is colored with a rainbow gradient which varies from the N-terminus (blue) to the C-terminus (red).

The putative alginate lyase, Alg169, is composed of 563 amino acids (Figure 2A). Alg169 showed a calculated molecular weight (MW) of approximately 62.6 kDa and a calculated isoelectric point (PI) of 3.92. This enzyme shares substantial sequence similarity with members of polysaccharide lyase family 7 (PL7) WP_290039577.1 (99.8%) and WP_2585546825.1 (95.0%), neither of which has been previously characterized. According to Conserved Domain search analyses conducted on NCBI, Alg169 possesses two catalytic domains, as shown in Figure 2A. Sequence alignment with other alginate lyases from the PL7 family categorizes Alg169 within subfamily 5 of the PL7 family (Figure 2B). The enzyme exhibits conserved sequence characteristics of PL7 family alginate lyases, which include motifs R*E*R, Q (I/V)H, and Y*KAG*Y*Q (Figure 2C) [34,35]. Amino acids His^138^, Arg^91^, Tyr^259^, and Gln^136^ are critically conserved within the N-terminal domain; His^432^, Arg^361^, Tyr^539^, and Gln^430^ remain strictly conserved in the C-terminal domain of the enzyme (Figure 2C). Alginate is a natural high-molecular polysaccharide containing mannuronic acid and guluronic acid. Structure prediction and molecular docking with alginate (Figure 3A), L-tetraguluronic acid (Figure 3B), and D-tetramannuronic acid (Figure 3C) revealed similar binding sites across substrates, involving conserved residues His^138^, Arg^91^, Tyr^259^, and Gln^136^ in the interaction. These results suggested that Alg169 functions as a bifunctional enzyme with completely different binding sites in the two domains.

### 3.3. Analysis of the Three-Dimensional Structure of Alg169

Based on crystal structures analysis of the PL7 family alginate lyases, Alg169 exhibits the highest identity (59.79%) with the alginate lyase from *Klebsiella pneumoniae* (PDB identifier: 4OZX). The prediction of the 3D structure of Alg169 was carried out using AlphaFold2 [32], with the structure quality validated by the SAVES v6.0 web platform. Analysis via Ramachandran plots revealed that the proportion of the residues located within the most favored regions, additionally allowed regions, and generously allowed regions are 88.4%, 10.4%, and 0.8%, respectively. And only 0.4% of residues were in the disallowed regions. The optimal model achieved an ERRAT score of 96.45 (Figure S2) for overall structural quality, and the Verify3D assessment yielded a score of 94.31% (Figure S3). These results showed that the 3D structure of Alg169 predicted by AlphaFold2 is of high quality [36].

**Figure 4:**
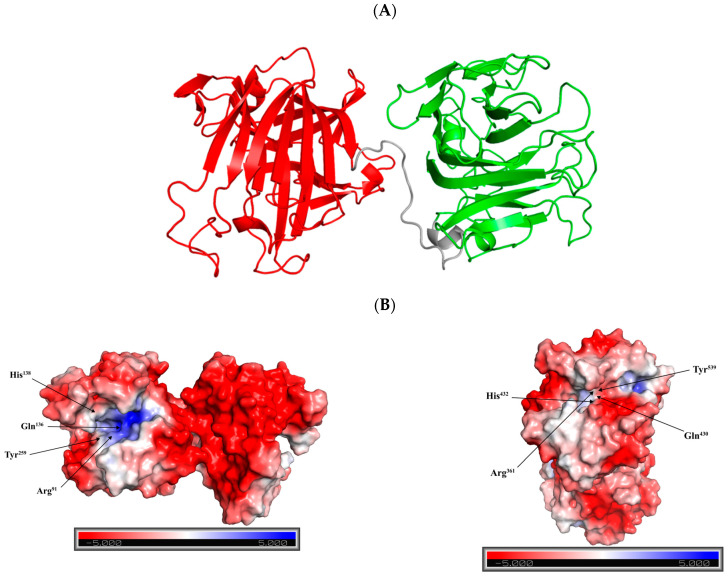
The 3D architecture of Alg169. (**A**) The dual-domain structure of Alg169, with the N-terminal domain colored red (covering residues 39 to 276) and the C-terminal domain in green (covering residues 296 to 559), separated by a linker region, which is colored grey. (**B**) The electrostatic surface analysis for the 3D structure of Alg169 is illustrated, pointing out critical residues within both domains that are positioned in positively charged gaps, indicated with black arrowheads. The illustration uses blue to denote areas of positive charge and red for negative areas.

Alg169 consists of two primary structural domains of the alginate lyase superfamily 2, and the two domains are linked by a flexible linker region (Figure 4A). Each domain is composed of four helices and two large *β*-sheets, Sheet A and Sheet B. Both Sheet A and Sheet B are formed from fifteen *β*-strands organized in an antiparallel configuration, together creating the enzyme’s core architecture. These *β*-strands together constitute the enzyme’s main structural framework. The catalytic sites are situated within the fissure created by the inner sheet A, where the alginate substrate engages with the catalytic residues, leading to the release of the reaction product from the enzyme’s central area [34]. The N-terminal domain spans residues 39 to 276, and the C-terminal domain extends from residues 296 to 559. Furthermore, analysis of the electrostatic surface revealed the key residues Arg^91^, Gln^136^, His^138^, and Tyr^259^ in the N-terminal domain situated in areas of positive charge, and similar observations were made for the C-terminal domain (Figure 4B). These areas are likely to contribute to forming a binding site for alginate substrates with negative electric charges. The cleavage of alginate is a process involving a *β*-elimination reaction, catalyzed by acid-base catalysis. The reaction occurs between the alginate molecule’s +1 and −1 sites, which necessitates the neutralization of the carboxylic group’s negative charge at the +1 position [34]. During the enzymatic degradation of sodium alginate, the negative charge on the carboxyl group is neutralized by arginine and glutamine. Histidine facilitates the removal of protons from C-5, while tyrosine donates a proton to break the 1,4-glycosidic linkage, resulting in the formation of a double bond [37].

### 3.4. Expression and Purification of Alg169

**Figure 5:**
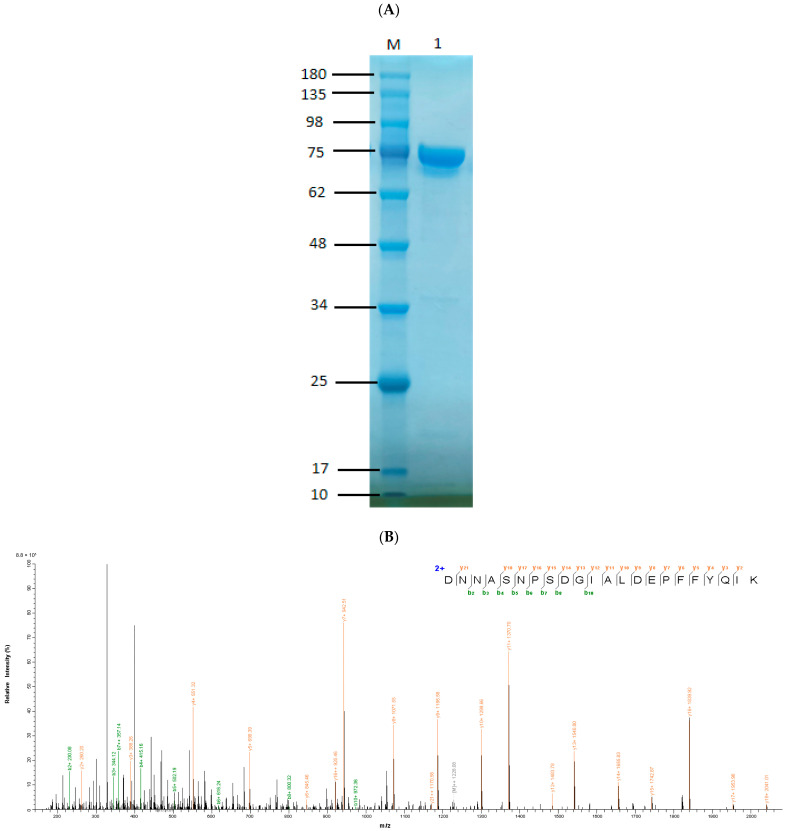
Protein purification and detection. (**A**) SDS-PAGE analysis of Alg169. Channel M, molecular weight marker (RuiBiotech, Beijing, China); Channel 1, purified Alg169 from induced cell lysate. (**B**) Peptide mass spectrum of Alg169 LC-MS/MS detection. The abscissa is the mass-to-charge ratio (*m*/*z*), and the ordinate is the signal intensity. The b and y ions are shown on the top. The green is b ion and the orange is y ion.

To obtain purified Alg169, a 6 × His tag sequence was added to the downstream of the Alg169 gene for subsequent purification. Then, the gene was cloned onto the pET-21a (+) vector, cloned, and expressed in *E. coli* BL21 (DE3). The expression of the alginate lyase Alg169 was induced by isopropyl-*β*-D-1-thiogalactopyranoside (IPTG). Then, the alginate lyase Alg169 was purified by His-tag Protein Purification IDA-Nickel magnetic beads and analyzed by SDS-PAGE (Figure 5A). LC-MS/MS verification predicted molecular weight of the Alg169 with tag protein was 65.58 kDa. The mass-spectrogram of a signature peptide of Alg169 is shown in Figure 5B. The genes *alg189*, *alg179*, and *alg136* were also cloned and expressed. After determining the activity of the crude enzyme, none of them were enzymatically active.

### 3.5. The Optimum Temperature and Temperature Stability of Alg169

**Figure 6:**
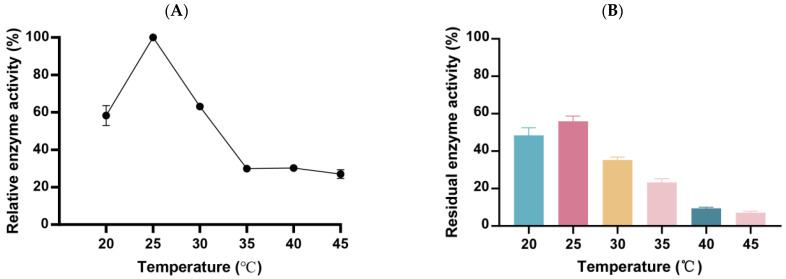
Effect of temperature on Alg169 activity. (**A**) Optimal temperature of Alg169. (**B**) temperature stability of Alg169. Each value represents the means of three replicates ± standard deviation.

The effect of different temperatures on the enzyme activity was investigated using 1% alginate as substrate. The optimum reaction temperature of alginate lyase Alg169 is 25 °C (Figure 6A). It maintained more than 50% of the highest enzyme activity at 20–30 °C. Temperature change has a significant effect on the enzyme activity of Alg169. The study of temperature stability showed that the enzyme activity in optimum temperature incubated 2 h only retained less than 60% enzyme activity of no-incubating (Figure 6B). The enzyme activity sharply declines once the temperature surpasses 25 °C and becomes inactive when the temperature reaches 45 °C.

### 3.6. The Optimum pH and pH Stability of Alg169

**Figure 7:**
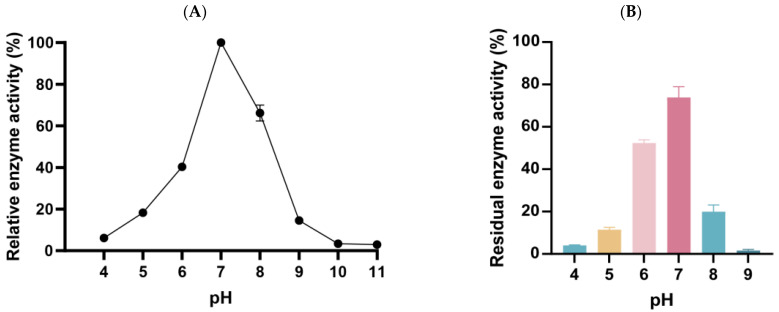
Effect of pH on Alg169 activity. (**A**) Optimal pH of Alg169. (**B**) pH stability of Alg169. Each value represents the means of three replicates ± standard deviation.

The effects of different pHs on the enzyme activity were investigated using 1% alginate as substrate. In the study of the optimum pH of the enzyme (Figure 7A), the optimum pH of the enzyme was 7.0 and the buffer system was 50 mmol/L Tris-HCl. The activity of the enzyme changed significantly with the pH modification. It showed that Alg169 is suitable for a neutral environment. By studying the effects of different pHs on the enzyme activity (Figure 7B), we found that different pHs had significant effects on enzyme activity, and enzyme activity decreased to less than 20% of the original enzyme activity in an acidic environment (pH 5.0–6.0) or an alkaline environment (pH 8.0–9.0). In strong acid and alkaline environments (pH 4.0 and 9.0), Alg169 was almost inactivated. In a neutral environment, the enzyme activity can be maintained at more than 70% of the original enzyme activity.

### 3.7. The Effect of Metal Ions on Alg169 Enzyme Activity

**Figure 8:**
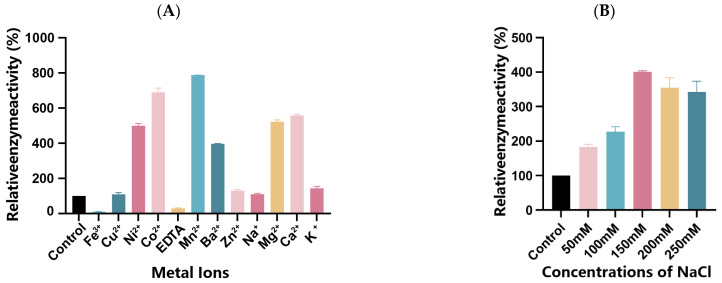
(**A**) Effect of different metal ions on Alg169 activity. (**B**) Effect of different concentrations of NaCl on the activity of Alg169. Each value represents the means of three replicates ± standard deviation.

Different kinds of metal ions were added to the reaction system with 1% sodium alginate to evaluate the effect of metal ions on enzyme activity. The final concentration of metal ions was 5 mmol/L and the enzyme activity without metal ions was defined by 100%. Most metal ions can enhance the activity of Alg169 (Figure 8A). Among the tested divalent cations, Mn^2+^, Co^2+^, Ca^2+^, Mg^2+^, Ni^2+^, and Ba^2+^ demonstrated a greater promotional effect on enzyme activity, with their effectiveness following the above sequence. In particular, the enzyme activity increased by 687.6% after Mn^2+^ treatment. Cu^2+^, Zn^2+^, Na^+^, and K^+^ increased the enzyme activity by 0.1–0.5 times, and Fe^3+^ and EDTA showed an inhibitory effect on Alg169.

### 3.8. The Effect of NaCl Concentration on the Activity of Alg169

To investigate the impact of NaCl concentration on enzyme activity, a reaction system containing 1% sodium alginate was supplemented with varying concentrations of NaCl and the enzyme, adding 5 mmol/L Mn^2+^. The control reaction involved only the addition of Mn^2+^ without NaCl. The results demonstrate that increasing NaCl concentration significantly enhances enzyme activity (Figure 8B). An increase in NaCl concentration from 50 to 150 mmol/L led to enhanced enzyme activity, with Alg169 enzyme activity increasing by 301.05% at 150 mmol/L, corresponding to a specific activity of 117,081.21 U/mg. Beyond a NaCl concentration of 150 mmol/L, the promotion effect on enzyme activity did not continue to increase and, in fact, slightly decreased. This observation suggests that the optimal NaCl concentration for Alg169 is 150 mmol/L.

### 3.9. Enzyme Kinetic Parameters and Products of Alg169

**Figure 9:**
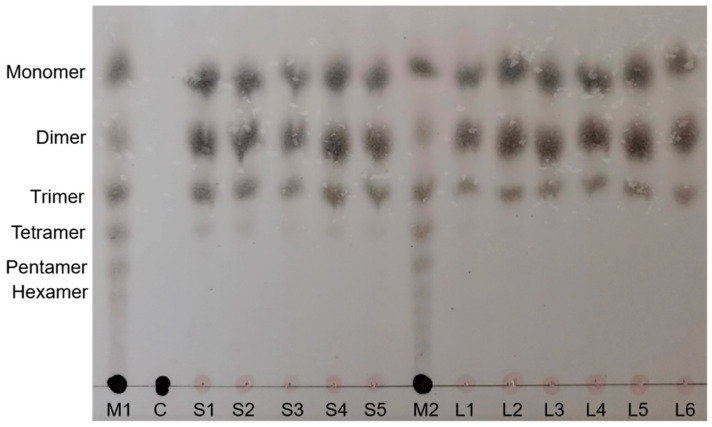
Thin-layer chromatogram (TLC) of alginate enzymatic hydrolysates. Alginate oligosaccharides 1–6 sugar standard sample (Channel M1, M2); 1% sodium alginate (Channel C); 1% alginate incubated with Alg169 for 1 min, 5 min, 10 min, 15 min, and 20 min (Channel S1–S5); 1% alginate incubated with Alg169 for 1 h, 6 h, 12 h, 24 h, 48 h, and 72 h (Channel L1–L6).

The kinetic parameters of the alginate lyase Alg169 were determined using varying concentrations of sodium alginate, polyG, and polyM as substrates, as shown in Table 1. Alg169 exhibited a lower Km value for polyG, suggesting a higher affinity for G-block substrates. Alginate oligosaccharides resulting from Alg169-mediated alginate degradation were primarily monosaccharides, disaccharides, and trisaccharides (Figure 9). Tetrasaccharides were briefly detected within 1 min of the reaction but were rapidly degraded and almost completely disappeared after 1 h, indicating that Alg169 has a bifunctional lyase activity and an efficient degradation rate. These observations are consistent with the molecular docking results and kinetic parameter analysis, further supporting Alg169’s efficiency in alginate degradation.

## 4. Discussion

Traditional approaches to discovering alginate lyases rely on the screening of alginate lyase-producing bacteria from environmental samples, followed by the cloning of the alginate lyase genes. However, these approaches are limited by the fact that some strains with potential alginate lyase activity may be unculturable under laboratory conditions, thus hindering the identification and characterization of novel enzymes [21]. In this study, we employed a metagenomic approach to identify alginate lyases from microorganisms present on the surface of refrigerated, decaying kelp. By using bacterial abundance as a screening criterion and integrating bioinformatics analysis, we successfully identified the alginate lyase gene *alg169*. Subsequently, we expressed, purified, and characterized the Alg169 enzyme to further assess its properties and potential applications.

Alg169 is a PL7 family alginate lyase member with two structural domains, the convergence of which allows for intramolecular synergy in alginate degradation, leading to the effective and thorough breakdown of diverse alginate substrates [38,39]. Alginate lyases featuring dual domains typically exhibit broader substrate specificity and offer enhanced potential for use in alginate degradation applications. Further investigation into the characteristics of these dual domains and an exploration of the synergistic effects between them can enhance our comprehension of the mechanisms underlying Alg169. This, in turn, could offer valuable insights for enzyme engineering aimed at optimizing Alg169 for the production of AOSs from alginate degradation.

The major characterization of Alg169 compared to other reported alginate lyases is listed in Table 2. The optimal pH and pH stability of Alg169 align with those of the majority of alginate lyases in the PL7 family, which demonstrate peak activity within a limited pH range and an optimal pH close to neutral [40]. Alg169 relies on ions for optimal activity and requires particularly either Mn^2+^ or Na^+^ to achieve high catalytic activity. It exhibited a notable advantage in enzyme activity under optimal conditions compared to other alginate lyases. The environmentally friendly reaction conditions (room temperature, neutral pH, and the presence of common ions) necessary for Alg169 to achieve high catalytic activity make it potentially suitable for industrial applications. Alg169 is an enzyme sourced from marine environments; as observed in numerous prior studies, enzymes from marine sources are often NaCl-dependent. Consequently, the anticipated result is the enhancement of its enzymatic activity with the addition of NaCl. Nevertheless, it is crucial to highlight that the optimal NaCl concentration for Alg169 activity is 150 mmol/L, which is slightly higher than the salt concentrations typically used in enzyme assays for most alginate lyases (ranging from 10 to 50 mmol/L). Notably, this concentration is similar to that of normal saline and should not hinder its potential for industrial applications. Additionally, the ions can be removed by dialysis for the purification of the produced oligosaccharides. Alg169 is sensitive to pH and temperature, which poses challenges for large-scale industrial applications. However, these limitations can be addressed and improved using enzyme engineering techniques, such as directed evolution.

Large-scale enzyme production is a critical prerequisite for the industrial application of Alg169. While culturing the natural producer is not currently feasible, heterologous overexpression in *E. coli* remains a viable option. Our study demonstrates the potential of heterologous overexpression in *E. coli* as a viable strategy. Notably, we achieved a commendable yield of 27.85 mg/L on a lab scale, even without optimization efforts. This finding suggests the significant potential for substantial enzyme overexpression using *E. coli* as the expression host. However, it is essential to acknowledge the limitations of *E. coli* for industrial applications, particularly in the food and beverage industry. Due to their Generally Recognized As Safe (GRAS) status, organisms like Bacillus subtilis and Pichia pastoris are more suitable choices for large-scale production of food-grade enzymes. Therefore, further studies will be focused on optimizing Alg169 production using these preferred expression systems.

Alg169 had lower K_m_ values and higher k_cat_/K_m_ values when it was incubated with polyM compared to sodium alginate and polyG. This indicates that Alg169 was more capable of catalyzing polyM than sodium alginate and polyG. Alginate lyase degrades alginate into AOS with the degree of polymerization (DP) 2–25 [47]. Most of the alginate lyases in the PL7 family mainly produce DP2–5 oligosaccharides in an endo-cleavage manner [28,48]. Alg169 displays a broad substrate specificity, and its hydrolysis of sodium alginate results in low-molecular-weight (MW) products with a relatively simple composition, mainly comprising DP1–3 oligosaccharides. This characteristic facilitates the subsequent purification processes. Previous studies suggest functional sugars with a lower degree of polymerization exhibit better bioavailability compared to highly polymerized functional sugars [47,49,50]. Consequently, Alg169 demonstrates potential advantages in producing low-molecular-weight oligosaccharides characterized by increased solubility and bioavailability.

In conclusion, Alg169 exhibits superior activity compared to a significant number of reported alginate lyases isolated from other sources. Notably, Alg169 functions effectively under mild conditions (room temperature, neutral pH, and presence of common ions). This reduces energy consumption and potential environmental hazards associated with harsh reaction conditions. These combined advantages position Alg169 as a promising candidate for industrial applications requiring efficient and environmentally friendly alginate degradation. While our approach does come with inherent limitations, including increased costs, ineffective screening methods for certain enzyme activities, and challenges related to heterologous expression systems [22,51,52], we were able to achieve a significant breakthrough by uncovering Alg169, an alginate lyase with remarkable enzyme activity. Future research will focus on enhancing its stability at varying pH and temperature ranges, exploring immobilization methods, and scaling up the degradation process for brown seaweed for industrial applications.

## Figures and Tables

**Table 1:** Kinetic parameters of Alg169 on sodium alginate.

Substrate	Sodium Alginate	polyG	polyM
K_m_ (mg/mL)	7.23	0.53	1.29
V_max_ (μg/min)	249.30	104.10	71.15
k_cat_ (s^−1^)	2678.63	2082.00	1423.00
k_cat_/K_m_ (s^−1^/(mg/mL))	370.49	3928.30	1103.10

**Table 2:** Comparison of the enzymatic properties of Alg169 with some other reported alginate lyases.

Alginate Lyase	Specific Activity (U/mg)	Optimal Temperature (°C)	pH	Cation Activators	Source	Reference
Alg169	117,081	25	7.0	Mn^2+^, Co^2+^, Ca^2+^, Mg^2+^, Ni^2+^, Ba^2+^, Cu^2+^, Zn^2+^, Na^+^, K^+^	*Psychromonas* sp. SP041	This study
AlyRm3	37,315	70	8.0	Na^+^, K^+^, Ca^2+^, Mg^2+^, Co^2+^, Fe^3+^	*Rhodothermus marinus* DSM 4252	[41]
FlAlyA	23,478	55	7.7	Na^+^, K^+^, Mg^2+^, Ca^2+^, EGTA	*Flavobacterium* sp. strain UMI-01	[42]
AlgA	8306	40	7.5	Na^+^, Mg^2+^, Ca^2+^	*Acillus weihaiensis* Alg07	[26]
AlyC3	6000	20	8.0	Na^+^	*Psychromonas* sp.C-3	[43]
AlgNJ-04	2416	40	7.0	Na^+^, K^+^, Ca^2+^	*Vibrio* sp. *NJ-04*	[28]
Aly2	2025	40	6.0	EDTA, glycerol, 2-mercaptoethanol	*Flammeovirga* sp. MY04	[44]
Alg4755	961	35	8.0	Al^3+^, Co^2+^, Ba^2+^, Fe^3+^, Ni^2+^, Na^+^	*Vibrio alginolyticus* S10	[45]
Algb	457	30	8.0	Na^+^, Ca^2+^, Co^2+^, Fe^2+^	*Vibrio* sp. W13	[46]

## Data Availability

The data presented in this study are available on reasonable request from the corresponding authors.

## Supplementary Materials

The following supporting information can be downloaded at: https://www.mdpi.com/article/10.3390/genes15050598/s1, Figure S1: Ramachandran plot of the predicted protein structure of Alg169; Figure S2: ERRAT detection results of Alg169 predicted protein structure; Figure S3: Verify3D detection results of Alg169 predicted protein structure; Table S1: Gene sequences of Alg169, Alg179, Alg189, and Alg136; Table S2: Alg169 amplification and identification primers.
