# Supplementary material for: Expression and Characterization of an Efficient Alginate Lyase from Psychromonas sp. SP041 through Metagenomics Analysis of Rotten Kelp
Source: Genes (Basel). 2024 May 8;15(5):598. doi: 10.3390/genes15050598 (PMC11121350; doi:10.3390/genes15050598)
Supplement: Supplementary file 1 [file genes-15-00598-s001.zip › genes-2975157-supplementary.pdf]

Supplementary Materials

Expression, purification and characterization of a new alginate lyase from *Psychromonas* sp. SP041 through metagenomics analysis of rotten kelp

Ping Wang[1, 2], Yi Cai[3], Hua Zhong[4], Ruiting Chen[3], Yuetao Yi[2], Yanrui Ye[3,*], Lili Li[2,*]

[1]Shandong University of Traditional Chinese Medicine, Jinan, 250355, China; wangpingdoct@163.com

[2]Yantai Institute of Coastal Zone Research, Chinese Academy of Sciences, Yantai, 264003, China; wangpingdoct@163.com; ytyi@yic.ac.cn; llli@yic.ac.cn

[3]South China University of Technology, School of Biology and Biological Engineering, Guangzhou, 510006, China; 202110189261@mail.scut.edu.cn; 202221049742@mail.scut.edu.cn; esyanruiye@scut.edu.cn

[4]Binzhou Medical University, Yantai, 264003, China; 18279017719@163.com

*Correspondence: esyanruiye@scut.edu.cn; llli@yic.ac.cn

Content

Figure S1, S2 and S3
Table S1
Table S2

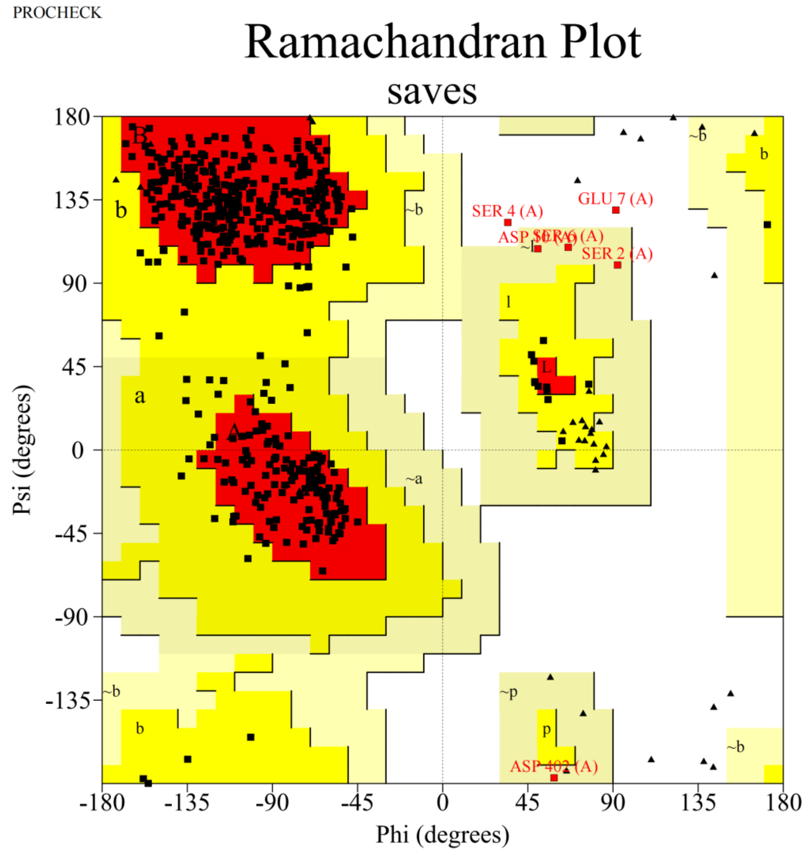

Plot statistics

Residues in most favoured regions [A,B,L]	444	88.4%
Residues in additional allowed regions [a,b,l,p]	52	10.4%
Residues in generously allowed regions [~a,~b,~l,~p]	4	0.8%
Residues in disallowed regions	2	0.4%
	----	------
Number of non-glycine and non-proline residues	502	100.0%
Number of end-residues (excl. Gly and Pro)	2	
Number of glycine residues (shown as triangles)	41	
Number of proline residues	17	

Total number of residues	562	

Based on an analysis of 118 structures of resolution of at least 2.0 Angstroms and R-factor no greater than 20%, a good quality model would be expected to have over 90% in the most favoured regions.

saves_01.ps

Figure S1. Ramachandran plot of the predicted protein structure of Alg169

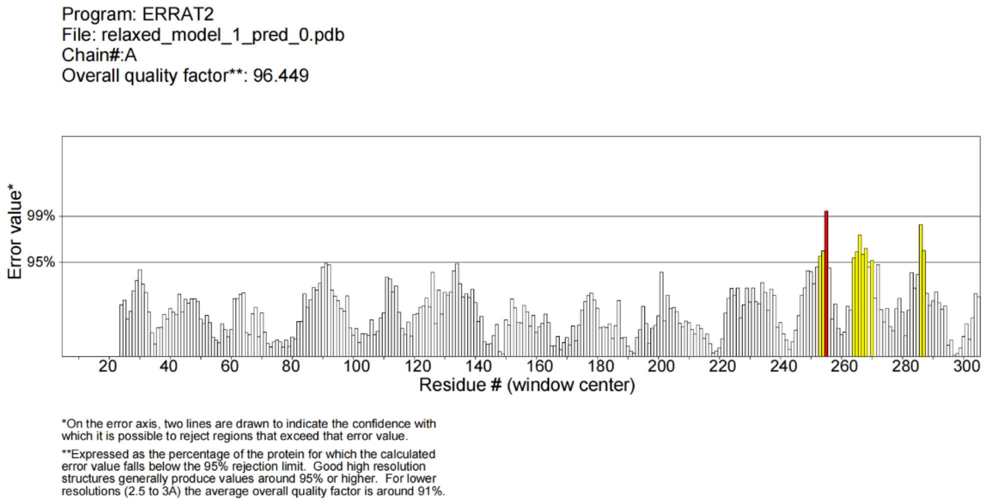

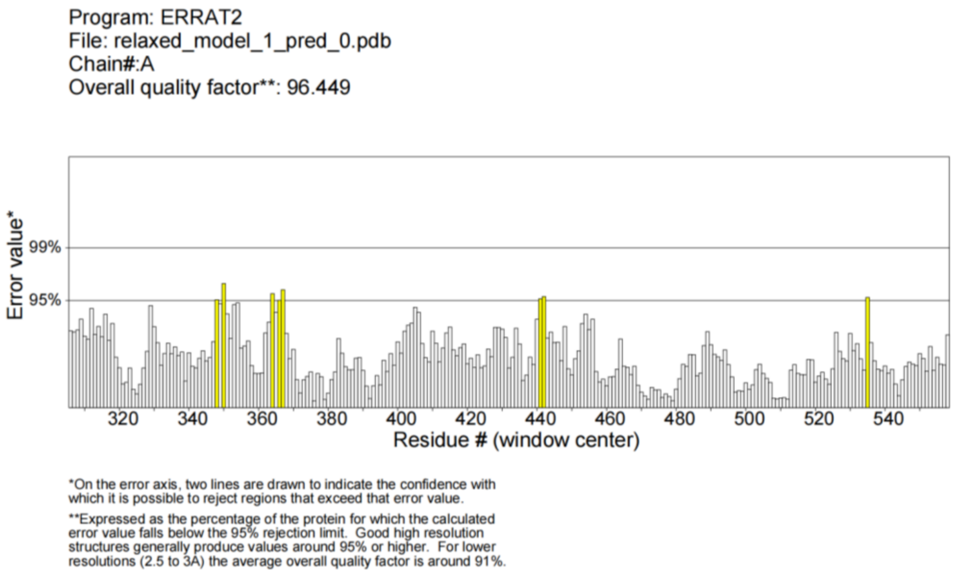

Figure S2. ERRAT detection results of Alg169 predicted protein structure

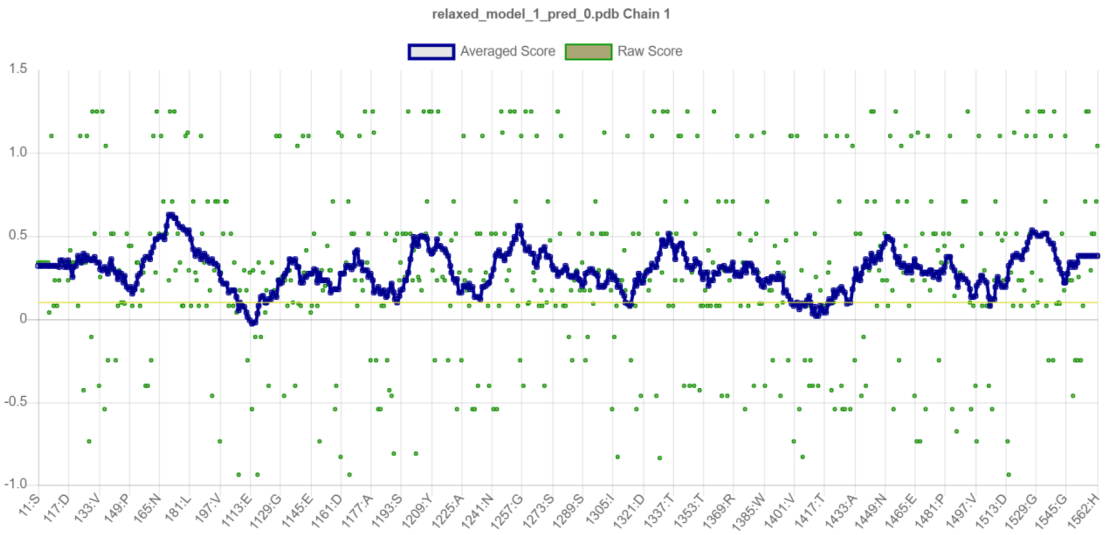

Figure S3. Verify3D detection results of Alg169 predicted protein structure

Table S1. Gene sequences of Alg169, Alg179, Alg189 and Alg136

Gene name	Gene sequence
Alg169	TCAAGCAGTTCATCCTCTGAAGGAACAGATACCGACTCCTCATCCTCTGACAACA<br>GCTCGTCGACCGGTTCCTACTCCGGCGTTCCGTATGATTATGTGCAATACATCCA<br>CGCCCTGAACAATGCCAAGCTGCAGCAAAACAACCCTCCGACCTCCAGCAGCAC<br>TTCAGAGGTGGTGGATGCTGGTGAGTACAACGGCTTTGACAACGACTACTTCTAC<br>CTGGATAGCACCACGGGTTGGCTGACCTTCGAAATGACCGGTGACTCGAAACGT<br>AGCGAGTTGCGCTTCGTAGAAAACTTTCGTACCAACACCTCAGAGATCAGCACG<br>CTTAGCGCGGAAATCTTACCGATTAGCCCGGAAGAAAGCGTTGCTAGCAGCGGT<br>GATGGGCAAGAGATGACGCTTATGCAGGTTCATAATAAAGGTGAGAATGGTGAA<br>ACGGATGATACCGTTCTGTCACATCCGCTGCTGCGTATTGTTTGGGATGGCGAGT<br>CTCGTATCGATGACGACACCGGTGACTCCTACTCTAACGCGTATTGGGCAATTAT<br>TAAGACGAATGCGTACGAATGTAAAGATGACAGCAACCCGAATTATAACGATAA<br>CTGCCCGGATAGTTACGATTTCTACTACCTGGCAGATTACGACCAGGATAACGCG<br>ACCAAGTTCGATATCGAAGCGGGTGACAGCACTCTGATTATCAACGTGGACGGT<br>GATGAGAAGGTGAACATTGATATTACCTATTGGGATGACCTCTATAGCTACTTCA<br>AGGCAGGCGTGTATAACCAGTACGAAGATGGTACCAGCACCGTTCAATTTAAGA<br>GCCTGACGTACAACGGCACCACCCTGCCGTCCGCGGACCTCGACTCTTCTTCTGC<br>GCCATCGGATAACTTCGAGTTGAATGATTGGAATCTGTCTATCCCGACGGACGAC<br>GACAACTCCGGTACCGCTGACACTATCGACGAGGATGACTTGAGTGACGGCTAC<br>GAAGACTTCGAATATTTCTATACCAATACCGACGACGGCGGCATGGTTTTTTACG<br>TGGAAGTCGACGGCTACAAAACCAGCACTAATACGAGCTATACCCGTACCGAGC<br>TGCGCGAGATGCTGAGAGAAGGTGACACCTCGATTAGCACTAAGGGTGTCAATG<br>AAAACAACTGGGTCTTCAGCAGTGCCCCGACTTCCGAGCAGGAGAAGGCGGGCG<br>GTGTGGACGGTATCCTGGACGCGACCGTTGCTGTGAATCAGGTGAGCACGACCG<br>GCGAGAGCTATCAAATCGGCCGTGTTATCATTGGCCAAATCCACGCTAATGACG<br>ACGAGCCGGTTCGCTTGTATTACCGCAAACTGCCGGACAACGATAAAGGTAGCA<br>TCTATTTCGCGCACGAACCGCGTACCGGCGATGAGGTCTGGGTTGATATGATCGG<br>TAGCCGTGATAATAACGCGTCCAACCCGAGCGACGGTATCGCGCTGGATGAGCC<br>ATTTTTTTATCAAATTAAAGTGGTTGGTAATACTTTGACCGTGACCATTATGCGT<br>GATGGCAAAGATGATGTTATTGAAACCTGGGACATGACGGATTCCGGCTATGAT<br>GAGACAGGCCAGTACATGTATTTTAAGGCGGGTGCCTACAACCAGAATAACAGC<br>GGTGAAGACGATGAAGCGGCACAGGCTACCTTTTATTACTTGAATAACCGCCAT
Alg179	CACGCTGATTATTTTTACCTAGACGATGAAGACCACCTGGTTTTTGTTACCCCGAA<br>CAAGGCGTTTACCACCCCGAACAGCTCTAATGCGCGTACGGAACTGCGTCAGATG<br>TTGCGCGGCACTGACACCTCCATTGGTACGCATGATCCGCAGAATAACTTCGCCTT<br>GGCGAGCCATAAACACGCAGACCAATACGCGCAGGTTGGCGGTTTTCTGGAAGCT<br>ACCCTCCGCGTCGAGCACGTGGCGGAACGTAGCCGTAAGCCGGACCGCAAGTCC<br>GCGTATTCGGTGGTGGTTGGTCAAATTCACGCCGGTAAAGATAAAGCTCTGATGG<br>AAGAGGACGAGGGCTTCGGCCATGGCAACGAGCCGCTGAAAATCTTCTACAAAA<br>AGCTTCCGGATCAAGATACCGGTAGCGTGTTCTGGAATTATGAGAAGAACCTGGC

GAAAGAAGACCCACGTCGTACCGACGTGAGCTACGCAGTTTGGGGTAATGATTGG
TCATCTAACGCTGATCCGGGTGAAGAGGGCATCAGCCTG

Alg189 GCTCACTATCTACCCCCACCTGATTTTATAAGAACACCGCCGGCAGCGCGTGCGCA
GCCGGCTGACTTCACCTGCCCGGCAGCTCCGGCGCCTTTCCGTGGCGAACTGGTT
TTTCGTTCGAAATACGAGGGCTCTGACGCTGCGCGAGCGACCTTGAATAAAGAAG
CACTCGCGGCGTACCGCCAGGCAACCCGCAACATCACGGCAACCCAAAAACGTG
TTACCGCGCTCGCTGCTCGCTATGGTAAGGCGGGCGATAAACGCGTGGCGGACTG
TCTGGTGAGCATGCTGTATGCCTGGGCGGTCCAGGATGCATTGACCAGCACCACC
ACCTCACATACCGGTAAGGCGGTTCGTAAGTGGGCGCTGGCATCCTTTAGCTCCG
CGTGGCTGCAATTGCAATTTGCCCCAGCCCAACCACTGAACGGCAACCCGCATAC
CGCGGAAGTTGAGCGCTGGCTGTCTGCATTGGCGACCCTGACTGTCTCTGACTGG
GCCGACCTGCCGCGTGAGAAGATTAACAACCACAGCTATTGGGCTGCGAGCGCCT
TAATGACCACTGCGGTCGTGACGGGTCGCGAGGATCTGTTCAACCGTGCGGTGGC
GATGCTAAGGACGGCTCTTTCCCAGGTTGATGAGAATGGTTTCCTGCCGAATGAAT
TAAAACGCAAAAGCCGTGCCCTGGCATACCACGATTACGCATTGCAACCGCTGGT
GACTTTGGTGCGTTTCGCGAAAGCGAATCAGGTTCGTCTGACGCCGGAGGAAAA
CCGCGCGTTTGCCCGTCTGGTGGCTCGTGTACGTGCTGGTAAGGCCGACCCGGCA
GCGTTTCGTGAAAAGACCGGCAGCGAACAGGAGCCGTTCACCCCGAGCCCGCGT
CCCTCCCCG

Alg136 ATGCTAACAAAAAAGACGCCCAATACTGTAACTACGAACAGTTGCAAGGTGGCA
GCGATTGCATGTAGCTTTGCCTTGCTGGGGTGCGTCTCTACGGTGGAAAACGATAC
CACCGTCCTGGCGAAAGCGGACAGCCCGGCGTCGCAATTTGATTTACTCGGCTGG
ACCATTTCTGTTCCGGTCGACTCCGACGGCAACGGCAAGTCCGATCAGATCAAAG
AAAAGGCTCTGGCTGCCGGTTATGCTGATCCGGAATACTTCTACTTGGCGGACGA
CGGCGGCATGGTTTTTAAATCTCCGGTTAAAGGCGCAAAAACGAGCAAAAACAC
CAAGTACACCAGAAGCGAGCTGCGTGAAATGATCCGCCGTGGTAATACCTCCTAC
AGCACCAAGGGCGTGGGTGGCAACAACTGGGTTTTCAGCACCGCACCGGCGGAA
GATCTGAAAAATGCAGGCGGCGTGGACGGCACCCTGGAAGCGACCCTTGCTGTG
AACCACGTTACCACCACTGGTGTGAATTGGCAGCAAGGTCGTGTTATTATCGGCC
AAATCCACGCCAATGATGATGAACCGATTCGCCTGTATTACCGCAAATTGCCACAA
CATGAGAAGGGTAGCATTTACTTCGCGCATGAGCCGAACTCGGAGGCGGACGAG
CAGTGGGTAGACATCATCGGTAACAGCCTGCCAAACTATTGGAATAAGGACGCTA
CACCGGCGGAGCCGGTCGACGGTATTAAACTGGGTGAAAAATTCTCCTACCGTAT
CAACGTGACGGGTCACGATTTGGCGGTGACTGTGATGCGTCCGGGTAAGGCCGA
CATCACCAAGCACGTGGACATGAGCGAGAGCCGTTATGATGTTGGTGGTCAATATA
TGTATTTCAAAGCGGGTGTTTATAACCAGAATAAGACCGGTGATGCGAAAGATTAC
GTTCAGGCAACCTTTTACGACCTGCAGGTTACGCAT

Table S2. Alg169 amplification and identification primers

Primer name	Primer sequence
Alg169-1	TTGTTTAACTTTAAGAAGGAGATATACATATGGCTAGCATGACTGGTGGACAGCAAATGGGTCGCGGATCCG
Alg169-2	GAGGATGAGGAGTCGGTATCTGTTCCTTCAGAGGATGAACTGCTTGAGAATTCGGATCCGCGACCCATTTGC
Alg169-3	CCGACTCCTCATCCTCTGACAACAGCTCGTCGACCGGTTCCTACTCCGGCGTTCCGTATGATTATGT
Alg169-4	GAGGGTTGTTTTGCTGCAGCTTGGCATTGTTCAGGGCGTGGATGTATTGCACATAATCATACGGAAC
Alg169-5	GCAGCAAAACAACCCTCCGACCTCCAGCAGCACTTCAGAGGTGGTGGATGCTGGTGAGTACAACGGCTTTGAC
Alg169-6	CGGTCATTTCGAAGGTCAGCCAACCCGTGGTGCTATCCAGGTAGAAGTAGTCGTTGTCAAAGCCGTTGTACTCAC
Alg169-7	TGACCTTCGAAATGACCGGTGACTCGAAACGTAGCGAGTTGCGCTTCGTAGAAAACTTTCGTACCAACAC
Alg169-8	CTTTCTTCCGGGCTAATCGGTAAGATTTCCGCGCTAAGCGTGCTGATCTCTGAGGTGTTGGTACGAAAGTTTTC
Alg169-9	TTAGCCCGGAAGAAAGCGTTGCTAGCAGCGGTGATGGGCAAGAGATGACGCTTATGCAGGTTCATAATA
Alg169-10	CAATACGCAGCAGCGGATGTGACAGAACGGTATCATCCGTTTCACCATTCTCACCTTTATTATGAACCTGCATAA
Alg169-11	ATCCGCTGCTGCGTATTGTTTGGGATGGCGAGTCTCGTATCGATGACGACACCGGTGACTCCTACTCTAAC
Alg169-12	TCATCTTTACATTCGTACGCATTCGTCTTAATAATTGCCCAATACGCGTTAGAGTAGGAGTCACCGG
Alg169-13	CGTACGAATGTAAAGATGACAGCAACCCGAATTATAACGATAACTGCCCGGATAGTTACGATTTCTACTAC
Alg169-14	CCGCTTCGATATCGAACTTGGTCGCGTTATCCTGGTCGTAATCTGCCAGGTAGTAGAAATCGTAACTAT
Alg169-15	CAAGTTCGATATCGAAGCGGGTGACAGCACTCTGATTATCAACGTGGACGGTGATGAGAAGGTGAACATTGATA
Alg169-16	TCGTACTGGTTATACACGCCTGCCTTGAAGTAGCTATAGAGGTCATCCCAATAGGTAATATCAATGTTCACCTT
Alg169-17	TGTATAACCAGTACGAAGATGGTACCAGCACCGTTCAATTTAAGAGCCTGACGTACAACGGCACCACCCT
Alg169-18	ATTCAACTCGAAGTTATCCGATGGCGCAGAAGAAGAGTCGAGGTCCGCGGACGGCAGGGTGGTGCCGTTGT
Alg169-19	TAACTTCGAGTTGAATGATTGGAATCTGTCTATCCCGACGGACGACGACAACTCCGGTACCGCTGACACTATCG
Alg169-20	TTGGTATAGAAATATTCGAAGTCTTCGTAGCCGTCACTCAAGTCATCCTCGT

	CGATAGTGTCAGCGGTACCG
Alg169-21	CGAATATTTCTATACCAATACCGACGACGGCGGCATGGTTTTTACGTGGAAGTCGACGGCTACAAA
Alg169-22	CTCTCAGCATCTCGCGCAGCTCGGTACGGGTATAGCTCGTATTAGTGCTGGTTTTGTAGCCGTCGACTT
Alg169-23	CTGCGCGAGATGCTGAGAGAAGGTGACACCTCGATTAGCACTAAGGGTGTCAATGAAAACAACTGGGTCT
Alg169-24	CAGGATACCGTCCACACCGCCCGCCTTCTCCTGCTCGGAAGTCGGGGCACTGCTGAAGACCCAGTTGTTTTCATT
Alg169-25	GGTGTGGACGGTATCCTGGACGCGACCGTTGCTGTGAATCAGGTGAGCACGACCGGCGAGAGCTATCAAATCGGC
Alg169-26	AGCGAACCGGCTCGTCGTCATTAGCGTGGATTTGGCCAATGATAACACGGCCGATTTGATAGCTCT
Alg169-27	GACGACGAGCCGGTTCGCTTGTATTACCGCAAACTGCCGGACAACGATAAAGGTAGCATCTATTT
Alg169-28	CACGGCTACCGATCATATCAACCCAGACCTCATCGCCGGTACGCGGTTCGTGCGCGAAATAGATGCTACCTT
Alg169-29	TGATATGATCGGTAGCCGTGATAATAACGCGTCCAACCCGAGCGACGGTATCGCGCTGGATGAGCCATTTTT
Alg169-30	CACGCATAATGGTCACGGTCAAAGTATTACCAACCACTTTAATTTGATAAAAAAATGGCTCATCCAGCGC
Alg169-31	CGTGACCATTATGCGTGATGGCAAAGATGATGTTATTGAAACCTGGGACATGACGGATTCCGGCTATGATGAG
Alg169-32	CGCTGTTATTCTGGTTGTAGGCACCCGCCTTAAAATACATGTACTGGCCTGTCTCATCATAGCCGGAAT
Alg169-33	TACAACCAGAATAACAGCGGTGAAGACGATGAAGCGGCACAGGCTACCTTTTATTACTTGAATAACCGCCATGCG
Alg169-34	GGGCTTTGTTAGCAGCCGGATCTCAGTGGTGGTGGTGGTGGTGCTCGAGTGCGGCCGCATGGCGGTTATTCAAGT
Pet21a-F	GATCCCGCGAAATTAATACGACTCAC
Alg169-R	GGTAGCCTGTGCCGCTTCAT

Note: Alg169 1-34 are amplification primers; Pet21a-F and Alg169-R are Alg169 bacterial PCR primers.
